# Health-related quality of life (HRQoL) after different axillary treatments in women with breast cancer: a 1-year longitudinal cohort study

**DOI:** 10.1007/s11136-023-03538-3

**Published:** 2023-10-27

**Authors:** N. J. M. C. Vrancken Peeters, Z. L. R. Kaplan, M. E. Clarijs, M. A. M. Mureau, C. Verhoef, T. van Dalen, O. Husson, L. B. Koppert

**Affiliations:** 1 Department of Surgical Oncology, Erasmus MC Cancer Institute, University Medical Center Rotterdam, 3015 GD Rotterdam, The Netherlands; 2 Department of Public Health, Erasmus MC, University Medical Center Rotterdam, Rotterdam, The Netherlands; 3 Department of Plastic & Reconstructive Surgery, Erasmus MC Cancer Institute, University Medical Center Rotterdam, Rotterdam, The Netherlands; 4 Department of Medical Oncology, Netherlands Cancer Institute-Antoni Van Leeuwenhoek, Amsterdam, The Netherlands

**Keywords:** Health-related quality of life (HRQoL), Patient-reported outcomes (PROs), Breast cancer, Axilla preserving surgery (APS), Axillary lymph node dissection (ALND), Axillary radiotherapy

## Abstract

**Purpose:**

As life expectancy continues to rise, post-treatment health-related quality of life (HRQoL) of breast cancer patients becomes increasingly important. This study examined the one-year longitudinal relation between axillary treatments and physical, psychosocial, and sexual wellbeing and arm symptoms.

**Methods:**

Women diagnosed with breast cancer who received different axillary treatments being axilla preserving surgery (APS) with or without axillary radiotherapy or full axillary lymph node dissection (ALND) with or without axillary radiotherapy were included. HRQoL was assessed at baseline, 6- and 12-months postoperatively using the BREAST-Q and the European Organization for Research and Treatment of Cancer QoL Questionnaire Breast Cancer Module (EORTC QLQ-BR23). Mixed regression models were constructed to assess the impact of axillary treatment on HRQoL. HRQoL at baseline was compared to HRQoL at 6- and at 12-months postoperatively.

**Results:**

In total, 552 patients were included in the mixed regressions models. Except for ALND with axillary radiotherapy, no significant differences in physical and psychosocial wellbeing were found. Physical wellbeing decreased significantly between baseline and 6- and 12-months postoperatively (*p* < 0.001, *p* = 0.035) and psychosocial wellbeing decreased significantly between baseline and 12 months postoperatively (*p* = 0.028) for ALND with axillary radiotherapy compared to APS alone. Arm symptoms increased significantly between baseline and 6 months and between baseline and 12 months postoperatively for APS with radiotherapy (12.71, 13.73) and for ALND with radiotherapy (13.93, 16.14), with the lowest increase in arm symptoms for ALND without radiotherapy (6.85, 7.66), compared to APS alone (*p* < 0.05).

**Conclusion:**

Physical and psychosocial wellbeing decreased significantly for ALND with radiotherapy compared to APS alone. Shared decision making and expectation management pre-treatment could be strengthened by discussing arm symptoms per axillary treatment with the patient.

**Supplementary Information:**

The online version contains supplementary material available at 10.1007/s11136-023-03538-3.

## Abstract (plain language summary)

Breast cancer is one of the most common cancers among women worldwide. Since the survival rates are improving, the health-related quality of life of breast cancer patients after treatment becomes more important. One of the most important factors in the prognosis of breast cancer patients is the presence of axillary metastasis, the spread of breast cancer to lymph nodes in the armpit. There are different axillary treatments which can all affect health-related quality of life negatively. Therefore, the aim of this study is to examine the effect of different axillary treatments on physical, psychosocial, and sexual wellbeing and arm symptoms of breast cancer patients. This study included women with breast cancer who received either axillary preserving surgery with or without axillary radiotherapy, or a full axillary lymph node dissection with or without axillary radiotherapy. health-related quality of life was assessed with validated breast cancer specific questionnaires at baseline, 6- and 12-months postoperatively. Patients who underwent axilla preserving surgery without axillary radiotherapy experienced significantly less decline in both physical and psychosocial wellbeing as well as significantly fewer increase in arm symptoms compared to patients who received the more extensive axillary treatments. The results of this study can be used to improve shared decision making and expectation management for future breast cancer patients.b

**Supplementary Information:**

The online version contains supplementary material available at 10.1007/s11136-023-03538-3.

## Background

Breast cancer is one of the most prevalent types of cancer among women, with an incidence of 16.000 new cases of invasive breast cancer per year in the Netherlands [1–3]. Due to the introduction of multiple novel therapies, the 10-year survival rate has gradually increased from 56 to 83% [4, 5]. Although the improvements in life expectancy are very promising, this also means that patients have to live with the possible negative consequences of breast cancer diagnosis and treatment which may affect their health-related quality of life (HRQoL), addressing the need for tailored treatment regimens [6, 7].

Axillary lymph node metastases are an important predictor of survival and recurrence in breast cancer patients and make treatment beyond the breast necessary [8–11]. In the early 90 s, the Sentinel Lymph Node Biopsy (SLNB) was introduced as a less invasive diagnostic for axillary lymph node staging in clinically node-negative (cN0) breast cancer patients [12]. If histopathological examination determined tumour-positive cells in the SLNB, several treatment options could follow, including an axillary lymph node dissection (ALND) and radiotherapy at different lymph node levels [13]. Treatment with ALND can be safely omitted in case of early stage breast cancer in a selection of patients who are treated with adjuvant chemotherapy or radiotherapy [14–17]. In comparison, clinically node-positive (cN +) patients were until recently, routinely treated with an ALND combined with or without axillary radiotherapy, also after neoadjuvant treatment, irrespective of their response to systemic treatment [13].

Axillary treatment with ALND and radiotherapy can cause significant upper extremity morbidities, such as disfunction of the arm, radiation fibrosis and lymphedema causing a decrease in HRQoL [18–20]. Recently, less invasive approaches for axillary treatment in cN + patients were introduced, known as Marking the Axilla with Radioactive Iodine seeds (MARI) procedure and Radioactive Iodine Seed Localization in the Axilla With the Sentinel Node (RISAS) procedure, a form of targeted axillary dissection [21–23]. The RISAS procedure is a combination of the SLNB and MARI procedure [22]. The marking procedure with iodine seeds is very elegant since, due to its long half lifetime, this marker stays radioactive during the months neo-adjuvant treatment is given. Additional treatment with ALND can be safely omitted when patients have a complete pathologic response according to the RISAS after neo-adjuvant treatment [21, 24]. Axillary preserving surgery has been associated with fewer complications compared to ALND and may, therefore, result in better HRQoL outcomes in breast cancer patients [16, 17, 25–27].

Previous studies have mainly focused on oncological outcomes of different axillary treatments in terms of overall survival or disease free survival [14–17, 28, 29]. Several studies have tried to investigate the effect of axillary treatment on HRQoL but lacked the inclusion of novel axillary preserving techniques or radiotherapy to the axilla. Additionally, the methodological quality shows wide variability with relative small sample sizes and lack the inclusion of baseline HRQoL measurements [26, 30–32]. Therefore, to enhance expectation management and shared decision-making, the objective of this study was to examine the longitudinal relation between different axillary treatments and HRQoL regarding the first year after surgery.

## Methods

This is a longitudinal cohort study of women with breast cancer who received different axillary treatments for lymph node staging and/ or metastasis at the Erasmus MC Cancer Institute in the Netherlands between November 1st, 2015, and January 1st, 2022. Inclusion criteria were women with a diagnosis of breast cancer who received axillary treatment and who completed Patient-Reported Outcome Measures (PROMs) in the “patient data platform”, Erasmus MC’s online PROM collection tool [33]. Exclusion criteria were patients who underwent proton therapy, palliative treatment, patients with recurrent breast cancer or if data on treatment were missing.

### Treatment

QoL was compared between four different axillary treatment groups, namely: [1] patients with axilla preserving surgery (APS) including SLNB and RISAS, [2] patients with an ALND (whether or not preceded by APS), [3] patients with APS followed by axillary radiotherapy and [4] patients with an ALND followed by axillary radiotherapy. In a SLNB, the median number of lymph nodes removed is 2. The RISAS procedure involves the removal of the marked lymph node metastasis plus the SLNB, which are in 71.3% the same lymph nodes. During an ALND usually a minimum of 10 lymph nodes are removed. The SLNB and RISAS procedure are combined in to one group since they only differ by a maximum of one lymph node in less than 30% of the cases.

Surgical treatment of the breast included either breast-conserving surgery (BCS) or mastectomy with or without reconstruction. Chemotherapy, hormonal therapy, and/ or (loco)regional radiotherapy were given to patients according to the national guidelines for breast cancer.

### Data-collection procedure

Data on sociodemographic factors, clinical treatment and tumour characteristics were retrospectively collected from medical records. These variables entailed, age at diagnosis, body mass index (BMI), gene mutation, presence of bilateral cancer, hormone receptor status, her2Neu receptor status and stage of disease at presentation (TNM staging system). Treatment characteristics entailed radiotherapy of the axilla and/or breast/ chest, parasternal radiotherapy, neo-adjuvant and/or adjuvant chemo(immuno)therapy, neo-adjuvant and/or adjuvant hormonal therapy, type of breast surgery and type of axillary treatment.

### HRQoL assessment

HRQoL data are routinely collected within the Erasmus MC Cancer Institute at baseline (first doctors visit; after diagnosis and before treatment initiation), at 6 months and at 12 months postoperatively according to the ICHOM Breast Cancer standard set [33, 34]. In this study, breast cancer specific questionnaires were used to assess HRQoL, namely the BREAST-Q and the EORTC QLQ-BR23 [35, 36]. In the statistical analysis only those domains in both questionnaires that are considered clinically the most relevant for comparing different axillary treatments were included to prevent the issues of multiple testing.

The BREAST-Q consists of two overarching themes, namely HRQoL and Patient Satisfaction. The HRQoL domains include psychosocial, sexual, and physical well-being. The satisfaction domains include satisfaction with breasts, outcome, and care. Scores of each domain range from 0–100 with higher scores representing better HRQoL and satisfaction. A minimal important difference score of 4 points was considered clinically relevant [35, 37]. Domains on psychosocial, physical, and sexual wellbeing from the BREAST-Q were included in the statistical analysis.

The EORTC QLQ-BR23 is a breast cancer-specific questionnaire consisting of 23 items. It incorporates functional scales and breast cancer specific symptom scales. Each item is scored on an ordinal scale: 1 = not at all, 2 = a little, 3 = quite a bit, 4 = very much [36]. According to the EORTC scoring manual, the EORTC QLQ-BR23 scores were linearly transformed to a numeric score (range 0–100) [36, 38]. For functional scales, higher scores represent a better level of functioning. Within the symptom-oriented scales, higher scores represent more severe symptoms. A minimal important difference score of 5 points on the transformed 0 to 100 scale was considered clinically relevant [38, 39]. The domain on arm symptoms from the EORTC QLQ-BR23 was included in the statistical analysis.

### Data analysis

Descriptive statistics, including frequencies and proportions, were used to describe patient, tumour, and treatment characteristics. Depending on the type of variable, ANOVA and Chi-square tests were used to compare the baseline characteristics of the four treatment groups. Means, standard deviations and percentages were calculated to present the results of HRQoL for each axillary treatment group. In addition, the percentages of missing values were also calculated. All scores were tested for normality with the Kolmogorov–Smirnov and Shapiro–Wilk tests. If normally distributed, parametric ANOVA tests were used to compare HRQoL between the pre-defined axillary treatment groups. If not normally distributed, non-parametric Kruskal–Wallis tests were used.

The longitudinal nature of the data, with repeated measurements within one patient (i.e., scores at baseline, 6- and 12- months postoperatively) induces correlation among these measurements and demands a multilevel approach. Therefore, to assess the effect of different axillary treatments on psychosocial wellbeing, physical wellbeing, sexual wellbeing, and arm symptoms, mixed effect linear regression was used. Patient was included as a random effect and the following fixed effects were adjusted for; age at diagnosis, BMI, type of breast surgery, radiotherapy of breast/chest, parasternal radiotherapy, neo-adjuvant hormonal therapy, neo-adjuvant chemo(immuno)therapy, postoperative hormonal therapy, and postoperative chemo(immuno)therapy. To avoid multicollinearity, tumour characteristics were not included as variables in the mixed models since they determine the treatment protocol. To analyse the impact of axillary treatment at different time points, an interaction term between time (baseline, 6 months, and 12 months) and axillary treatment was included in the model. APS without additional axillary radiotherapy was chosen as reference group since this is the least extensive axillary treatment. The effect of the different axillary treatments, between the different time periods, on the predefined outcomes was expressed as the delta. Delta 1 represents the change in HRQoL from the baseline measurement to 6 months postoperatively, and delta 2 represents the change in HRQoL from baseline to 12 months postoperatively. The delta gives more intuitive information compared to the crude measurements as it represents the relative effect of treatment on HRQoL. Positive deltas in BREAST-Q domains indicate an improvement in HRQoL, negative deltas a decline in HRQoL. Conversely, positive deltas in the arm symptoms domain of the EORTC QLQ-BR23 reflect an increase in arm symptoms and negative deltas a reduction in arm symptoms since this is a symptom orientated domain.

### Non-responder analysis

Because not all patients in the patient data platform completed all PROMs, a non-responder analysis was conducted to examine possible significant differences between responders and non-responders. Student’s unpaired t-tests and Chi-square tests were used to compare baseline characteristics. For all statistical analyses, a two-sided p-value of 5% was considered statistically significant. Statistical analyses were done using R statistical software (version 4.4.2) [40].

### Sample size calculation

As this study is a secondary analysis on a healthcare registration process, a sample size calculation is not applicable in this context.

## Results

### Patient characteristics

**Fig. 1:**
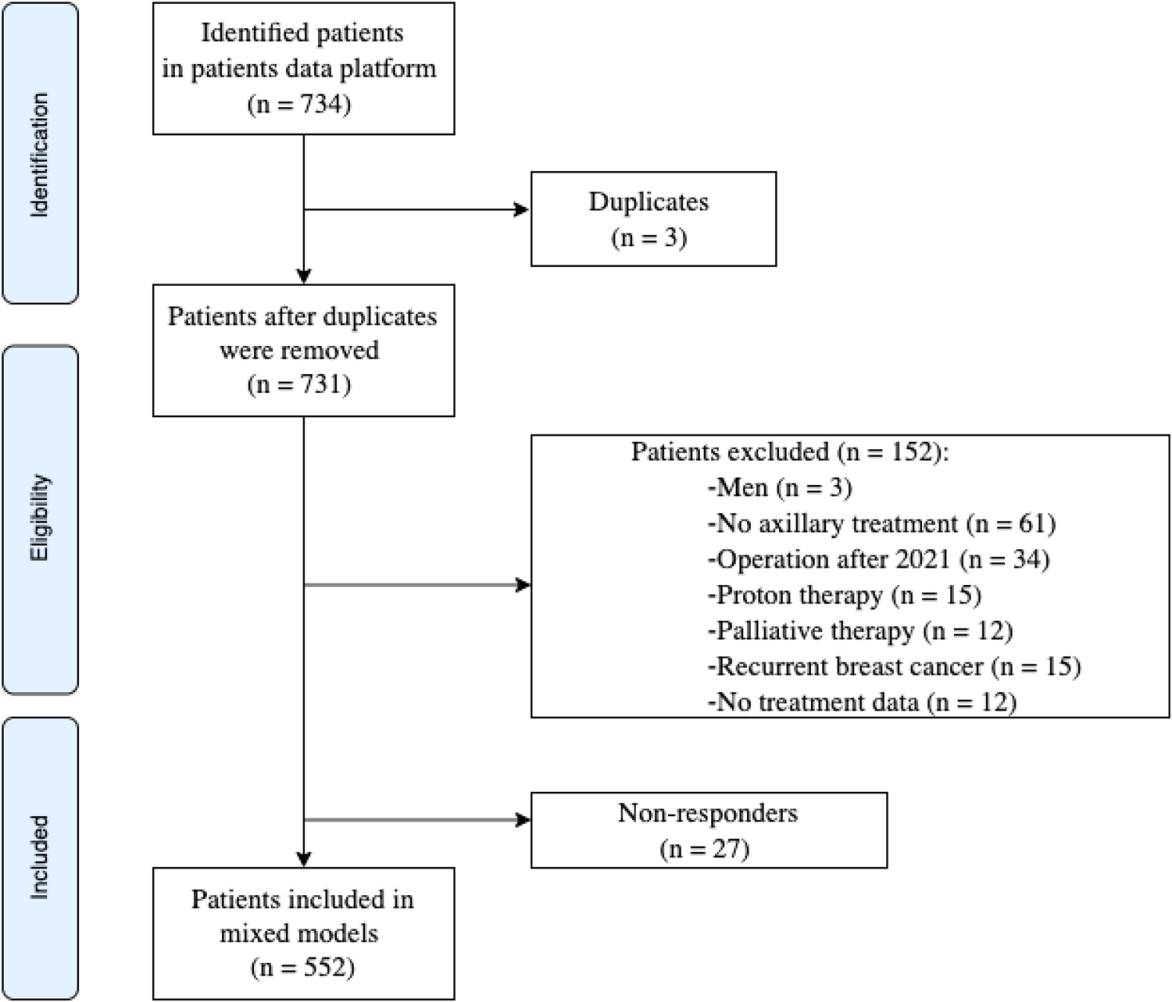
Flowchart

A total of 734 breast cancer patients was included from the institutional database, of whom 731 patients remained after duplicates were removed. Of those patients, 152 were excluded because they did not meet the inclusion criteria. Another 27 non-responders (< 5%) did not fill out the PROMs and were excluded, leaving 552 patients for the analyses (Fig. 1).

Of the 552 included patients, 386 patients (69.9%) underwent APS, 59 patients (10.7%) an ALND, 39 patients (7.1%) APS in combination with axillary radiotherapy and 68 (12.3%) patients underwent an ALND in combination with axillary radiotherapy (Table 1).

**Table 1:** Baseline characteristics according to treatment group

Characteristics	APS^*a*^ (*n* = 386)	ALND (*n* = 59)	APS^*a*^ + Rtx^*a*^ (*n* = 39)	ALND + Rtx^*a*^ (*n* = 68)	*p*-value
*Age*					
Mean (SD)	55.5 (14.1)	50.9 (15.3)	50.0 (13.5)	48.4 (12.8)	** < 0.001**
*BMI (%)*					
< 25	169 (43.8%)	30 (50.8%)	21 (53.8%)	35 (51.5%)	0.313
25–30	161 (41.7%)	18 (30.5%)	14 (35.9%)	20 (29.4%)	
> 30	56 (14.5%)	11 (18.6%)	4 (10.3%)	13 (19.1%)	
*Gene mutation (%)*					
No mutation	128 (33.2%)	33 (55.9%)	17 (43.6%)	41 (60.3%)	** < 0.001**
BRCA 1 mutation	35 (9.1%)	0 (0%)	3 (7.7%)	2 (2.9%)	
BRCA 2 mutation	20 (5.2%)	3 (5.1%)	0 (0%)	4 (5.9%)	
Other gene mutation	8 (2.1%)	2 (3.4%)	0 (0%)	1 (1.5%)	
Unknown	195 (50.5%)	21 (35.6%)	19 (48.7%)	20 (29.4%)	
*Bilateral cancer (%)*					
Yes	15 (3.9%)	8 (13.6%)	0 (0%)	4 (5.9%)	**0.006**
No	371 (96.1%)	51 (86.4%)	39 (100%)	64 (94.1%)	
*Surgery (%)*					
Mastectomy	77 (19.9%)	31 (52.5%)	8 (20.5%)	45 (66.2%)	** < 0.001**
BCS^***a***^	211 (54.7%)	21 (35.6%)	27 (69.2%)	19 (27.9%)	
Mastectomy + reconstruction	98 (25.4%)	7 (11.9%)	4 (10.3%)	4 (5.9%)	
*Receptor (%)*					
Triple negative	57 (14.8%)	4 (6.8%)	7 (17.9%)	17 (25.0%)	** < 0.001**
HER2 positive^***b***^	28 (7.3%)	9 (15.3%)	4 (10.3%)	17 (25.0%)	
HR^***a***^ positive & HER2 negative	258 (66.8%)	46 (78.0%)	28 (71.8%)	34 (50.0%)	
Unknown	43 (11.1%)	0 (0%)	0 (0%)	0 (0%)	
*T-stage (%)*					
pT0	24 (6.2%)	6 (10.2%)	6 (15.4%)	17 (25.0%)	** < 0.001**
pT1	242 (62.7%)	27 (45.8%)	24 (61.5%)	18 (26.5%)	
pT2	69 (17.9%)	19 (32.2%)	9 (23.1%)	13 (19.1%)	
pT3	3 (0.8%)	5 (8.5%)	0 (0%)	9 (13.2%)	
pT4	0 (0%)	0 (0%)	0 (0%)	2 (2.9%)	
pTis	48 (12.4%)	2 (3.4%)	0 (0%)	9 (13.2%)	
*N-stage (%)*					
pN0	378 (97.9%)	13 (22.0%)	6 (15.4%)	25 (36.8%)	** < 0.001**
pN1	8 (2.1%)	45 (76.3%)	33 (84.6%)	17 (25.0%)	
pN2	0 (0%)	1 (1.7%)	0 (0%)	19 (27.9%)	
pN3	0 (0%)	0 (0%)	0 (0%)	7 (10.3%)	
*Neoadjuvant CTx*^***a***^ *(%)*					
No	328 (85.0%)	32 (54.2%)	20 (51.3%)	17 (25.0%)	** < 0.001**
Yes	58 (15.0%)	27 (45.8%)	19 (48.7%)	51 (75.0%)	
*Adjuvant CTx*^***a***^ *(%)*					
No	324 (83.9%)	36 (61.0%)	23 (59.0%)	33 (48.5%)	** < 0.001**
Yes	62 (16.1%)	23 (39.0%)	16 (41.0%)	35 (51.5%)	
*Neoadjuvant HTx*^***a***^ *(%)*					
No	373 (96.6%)	54 (91.5%)	39 (100%)	65 (95.6%)	0.149
Yes	13 (3.4%)	5 (8.5%)	0 (0%)	3 (4.4%)	
*Adjuvant HTx*^***a***^ *(%)*					
No	219 (56.7%)	11 (18.6%)	9 (23.1%)	25 (36.8%)	** < 0.001**
Yes	167 (43.3%)	48 (81.4%)	30 (76.9%)	43 (63.2%)	
*Breast/ chest Rtx*^***a***^ *(%)*					
No	174 (45.1%)	21 (35.6%)	0 (0%)	0 (0%)	** < 0.001**
Yes	209 (54.1%)	35 (59.3%)	37 (94.9%)	50 (73.5%)	
Yes & parasternal	3 (0.8%)	3 (5.1%)	2 (5.1%)	18 (26.5%)	
*Rtx*^***a***^* boost (%)*					
No	302 (78.2%)	41 (69.5%)	23 (59.0%)	49 (72.1%)	**0.032**
Yes	84 (21.8%)	18 (30.5%)	16 (41.0%)	19 (27.9%)	

Bold values indicate the *p*-value < 0.05

^***a***^APS, axilla preserving surgery; Rtx, radiotherapy; BCS, breast conserving surgery; HR, hormone receptor; CTx, chemo(immune)therapy; HTx, hormonal therapy

^***b***^HR + /HR- combined

Most baseline characteristics, except for BMI and neo-adjuvant hormonal therapy, demonstrated significant differences between treatment groups. Patients who underwent ALND were more likely to have undergone a mastectomy, had a higher tumour stage, and received systemic therapy more frequently (Table 1).

### Non-responder analysis

The number of non-responders in the study was 27 (< 5%). The non-responder analysis showed similar distributions between responders and non-responders for almost all variables, except for BMI category. Non-responders had a higher BMI (BMI > 30) compared to responders (*p* = 0.008). The full non-responder analysis can be found in Apendix 1.

### HRQoL scores according to axillary treatment

There were no statistically significant differences in sexual wellbeing scores between treatment groups (Table 2). However, physical wellbeing scores differed significantly between treatment groups at 6 months postoperatively (*p* < 0.001, Table 2). Additionally, the psychosocial wellbeing domain also showed significant differences between treatment groups at 6 months (*p* = 0.010) and 12 months postoperatively (*p* = 0.012, Table 2). Furthermore, at 6- and 12-months postoperatively arm symptoms differed significantly between treatment groups (both *p* < 0.001, Table 2). HRQoL scores of other domains of the BREAST-Q and EORTC QLQ-BR23 can be found in Appendix 2.

**Table 2:** BREAST-Q and EORTC QLQ-BR23 scores on all follow-up times according to treatment group

HRQoL	APS	ALND	APS + Rtx	ALND + Rtx	*p*-value
	(*n* = 386)	(*n* = 59)	(*n* = 39)	(*N* = 68)	
BREAST-Q: Physical wellbeing at baseline					
Mean (SD)	80.9 (15.2)	78.1 (14.8)	82.1 (15.4)	78.6 (17.8)	0.520
Missing *(%)*	89 (23.1%)	15 (25.4%)	7 (17.9%)	14 (20.6%)	
BREAST-Q: Physical wellbeing at 6 months					
Mean (SD)	69.4 (18.1)	66.8 (24.4)	67.1 (16.3)	57.8 (18.5)	** < 0.001**
Missing *(%)*	99 (25.6%)	12 (20.3%)	11 (28.2%)	16 (23.5%)	
BREAST-Q: Physical wellbeing at 12 months					
Mean (SD)	71.9 (20.4)	66.0 (26.7)	70.7 (21.2)	65.3 (20.6)	0.145
Missing *(%)*	87 (22.5%)	9 (15.3%)	8 (20.5%)	16 (23.5%)	
BREAST-Q: Psychosocial wellbeing at baseline					
Mean (SD)	74.2 (17.5)	70.1 (19.0)	75.2 (17.6)	72.3 (20.1)	0.510
Missing *(%)*	89 (23.1%)	15 (25.4%)	7 (17.9%)	14 (20.6%)	
BREAST-Q: Psychosocial wellbeing at 6 months					
Mean (SD)	69.8 (18.0)	61.9 (16.5)	63.8 (15.2)	64.0 (20.7)	**0.010**
Missing *(%)*	170 (44.0%)	19 (32.2%)	18 (46.2%)	22 (32.4%)	
BREAST-Q: Psychosocial wellbeing at 12 months					
Mean (SD)	68.9 (19.5)	61.3 (18.1)	65.1 (18.7)	62.0 (21.3)	**0.012**
Missing *(%)*	164 (42.5%)	14 (23.7%)	15 (38.5%)	21 (30.9%)	
BREAST-Q: Sexual wellbeing at baseline					
Mean (SD)	63.7 (20.9)	64.3 (23.0)	68.5 (17.4)	64.3 (21.3)	0.729
Missing *(%)*	151 (39.1%)	29 (49.2%)	15 (38.5%)	22 (32.4%)	
BREAST-Q: Sexual wellbeing at 6 months					
Mean (SD)	48.3 (17.8)	45.4 (17.3)	49.6 (12.3)	49.4 (15.7)	0.729
Missing *(%)*	176 (45.6%)	28 (47.5%)	22 (56.4%)	32 (47.1%)	
BREAST-Q: Sexual wellbeing at 12 months					
Mean (SD)	50.3 (19.6)	46.3 (18.7)	48.6 (16.4)	49.1 (21.0)	0.739
Missing *(%)*	171 (44.3%)	21 (35.6%)	21 (53.8%)	30 (44.1%)	
EORTC QLQ_BR2^**a**^ 3: Arm symptoms at baseline					
Mean (SD)	8.65 (15.8)	7.41 (13.6)	5.05 (7.90)	8.05 (16.0)	0.844
Missing *(%)*	84 (21.8%)	14 (23.7%)	6 (15.4%)	10 (14.7%)	
EORTC QLQ_BR23^**a**^: Arm symptoms at 6 months					
Mean (SD)	13.2 (17.4)	19.0 (22.3)	22.6 (18.5)	26.6 (20.1)	** < 0.001**
Missing *(%)*	88 (22.8%)	11 (18.6%)	8 (20.5%)	12 (17.6%)	
EORTC QLQ_BR23^**a**^: Arm symptoms at 12 months					
Mean (SD)	12.8 (17.2)	19.4 (21.6)	22.2 (18.9)	27.3 (20.7)	** < 0.001**
Missing *(%)*	73 (18.9%)	8 (13.6%)	7 (17.9%)	15 (22.1%)	

Bold values indicate the *p*-value < 0.05

Symptom-oriented scales; higher scores represent greater symptom severity

^***a***^Functional scales; higher scores represent a better level of functioning

### Effect of axillary treatments on HRQoL over time

**Fig. 2:**
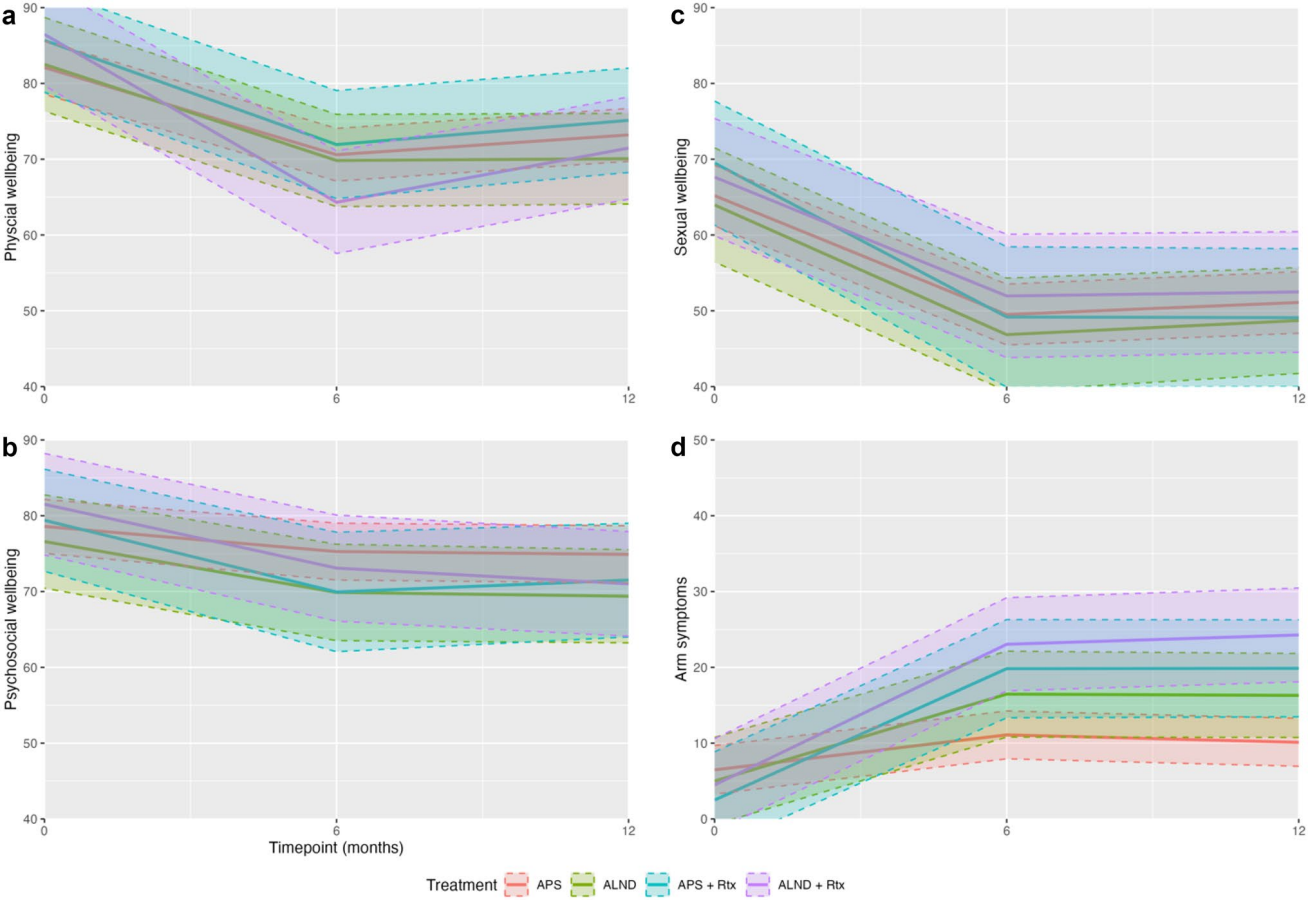
Effect plots of HRQoL over time according to axillary treatment group with the corresponding confidence intervals. The following characteristics were used in the effect plots: mean age, BMI < 25, BCS, adjuvant hormonal therapy and radiotherapy of the breast. Red represents APS, green ALND, blue APS + Rtx and purple ALND + Rtx. **a** Physical wellbeing; **b** Psychosocial wellbeing; **c** Sexual wellbeing; **d** Arm symptoms

HRQoL measured at baseline did not significantly differ between the more extensive axillary treatment groups (APS with axillary radiotherapy, ALND and ALND with axillary radiotherapy) compared to the APS group (reference) after adjusting for all variables (Table 3). For patients who underwent APS, physical wellbeing, psychosocial wellbeing, and sexual wellbeing decreased, and arm symptoms increased significantly at 6- and 12 months postoperatively (*p* < 0.05, Table 3, Fig. 2).

**Table 3:** Mixed model regression analysis

	Physical wellbeing		Psychosocial wellbeing		Sexual wellbeing		Arm symptoms	
	Estimates	*p*-value	Estimates	*p*-value	Estimates	*p*-value	Estimates	*p*-value
APS: baseline (intercept)	66.29	** < 0.001**	61.60	** < 0.001**	67.30	** < 0.001**	18.78	** < 0.001**
APS: 6 months postoperatively	– 11.50	** < 0.001**	– 3.32	**0.010**	– 15.68	** < 0.001**	4.62	** < 0.001**
APS: 12 months postoperatively	– 8.88	** < 0.001**	– 3.69	**0.004**	– 14.09	** < 0.001**	3.64	**0.002**
APS + Rtx	3.58	0.315	0.81	0.818	4.30	0.316	– 3.98	0.230
ALND	0.42	0.892	– 1.99	0.514	– 1.23	0.744	– 1.48	0.610
ALND + Rtx	4.37	0.193	2.92	0.380	2.44	0.526	– 1.98	0.516
APS + Rtx: delta 1^a^	– 2.24	0.541	– 6.14	0.142	– 4.62	0.356	12.71	**0.001**
APS + Rtx: delta 2^b^	– 1.66	0.644	– 4.20	0.300	– 6.31	0.199	13.74	** < 0.001**
ALND: delta 1^a^	– 1.18	0.703	– 3.40	0.313	– 1.42	0.734	6.85	**0.030**
ALND: delta 2^b^	– 3.56	0.239	– 3.53	0.276	– 1.15	0.773	7.66	**0.014**
ALND + Rtx: delta 1^a^	– 10.64	** < 0.001**	– 5.10	0.104	0.01	0.998	13.93	** < 0.001**
ALND + Rtx: delta 2^b^	– 6.11	**0.035**	– 6.81	**0.028**	– 1.05	0.774	16.14	** < 0.001**

Bold values indicate the *p*-value < 0.05

^a^Change in HRQoL from baseline to 6 months postoperatively

^b^Change in HRQoL from baseline to 12 months postoperatively

For patients who received APS with axillary radiotherapy, delta 1 (i.e., the difference between baseline and 6 months postoperatively) and delta 2 (i.e., the difference between baseline and 12 months postoperatively) of the arm symptoms domain increased significantly with 12.71 and 13.74 points respectively compared to patients undergoing APS alone (*p* < 0.05, Table 3, Fig. 2d). Similarly, for patients who underwent ALND, delta 1 (6.85) and delta 2 (7.66) of the arm symptoms domain increased significantly compared to patients undergoing APS alone (*p* < 0.05, Table 3, Fig. 2d). This indicates that arm symptoms increased significantly over time for patients undergoing APS with axillary radiotherapy and for patients undergoing ALND compared to patients undergoing APS alone. Furthermore, the increase in arm symptoms is greater for patients receiving APS with axillary radiotherapy than for patients receiving ALND. There were no significant differences in other BREAST-Q domains (Table 3, Fig. 2).

For patients who received ALND combined with axillary radiotherapy, negative delta 1 of the physical wellbeing domain increased with 10.64 significantly compared to patients undergoing APS (*p* < 0.001, Table 3, Fig. 2a). The corresponding negative delta 2 increased significantly with 6.11 points (*p* = 0.035, Table 3, Fig. 2a). Moreover, negative delta 2 of the psychosocial wellbeing domain increased with 6.81 points significantly for patients receiving ALND with axillary radiotherapy compared to patients undergoing APS (*p* = 0.028, Table 3, Fig. 2b). This indicates that the negative effect of axillary treatment on physical and psychosocial wellbeing increased significantly for patients undergoing ALND with axillary radiotherapy compared to patients undergoing APS alone. In addition, delta 1 (13.93) and delta 2 (16.14) of the arm symptoms domain increased significantly compared to patients undergoing APS (*p* < 0.001, Table 3, Fig. 2d). Sexual wellbeing did not significantly differ compared to APS (Table 3, Fig. 2c).

The effect plots demonstrate a decline in HRQoL from baseline to 6 months postoperatively. However, between 6 and 12 months postoperatively, HRQoL tends to stabilize again. The red colour represents the APS group, green the ALND group, blue the APS with axillary radiotherapy group and purple the ALND with axillary radiotherapy group (Fig. 2).

## Discussion

This longitudinal cohort study of women with breast cancer aimed to investigate the association between different axillary treatments and HRQoL measured with the BREAST-Q and the EORTC QLQ-BR23 in the first year after surgery. The results demonstrated that all axillary treatments included in this study led to an increase in arm symptoms. Moreover, APS with axillary radiotherapy and ALND with and without axillary radiotherapy led to a significant greater increase in arm symptoms compared to APS alone. In addition, the most extensive axillary treatment consisting of ALND combined with axillary radiotherapy had also a clinically significant effect on physical and psychosocial wellbeing. This indicates that ALND with axillary radiotherapy affects not only arm morbidity, but also more general HRQoL domains. This may be due to the potential physical and emotional side effects associated with axillary treatments such as pain, emotional distress, anxiety, and uncertainty about the long-term effects of the treatment [41, 42]. No significant difference in sexual wellbeing were found between the different axillary treatment groups.

The results of this study are consistent with some previous studies that have demonstrated benefits of SLNB compared to ALND [26, 31, 32, 43–47]. For instance, Belmonte et al. reported less arm morbidity in patients receiving SLNB compared to ALND which supports the use of SLNB. However, they did not find a significant difference in general well-being measured with the disease-specific FACT-B + 4 and the generic short form 36 health survey between ALND and SLNB [31]. Similarly, Peintiger et al. showed that SLNB is associated with significant less arm and shoulder morbidity compared to ALND. In addition, the study demonstrated significant higher levels of pain on the symptom scale of the EORTC QLQ-C30 in the ALND group [32]. Moreover, the prospective cohort study of Dabakuyo et al. showed that patients undergoing SLNB score better on both the global health status from the EORTC QLQ-C30, and the arm symptoms from the EORTC QLQ-BR23 compared to patients undergoing ALND [26]. A more recent study of Appelgren et al. also demonstrated that arm morbidity is significantly worse in patients receiving ALND compared to SLNB and highlights the importance of de-escalation of axillary surgery [47]. In contrast, Kootstra et al. did not demonstrate any association between SLNB and a better HRQoL measured with the EORTC QLQ-C30 compared to ALND [30]. Several randomized controlled trials have been conducted and all reported better HRQoL with fewer postoperative complications in patients undergoing SLNB compared to patients undergoing ALND [43–46]. The discrepancies between previous HRQoL studies can be explained by differences in sample size and by the fact that different validated questionnaires were used to measure HRQoL.

The AMAROS trial demonstrated that axillary radiotherapy may be a feasible alternative to ALND in cN + breast cancer patients, with comparable axillary control and less morbidity. However, the AMAROS did not demonstrate significant differences in HRQoL measured with the pain domain of the EORTC QLQ-C30 and the arm symptoms and body image domain of the EORTC QLQ-BR23 [17]. An interesting finding of the current study is that arm symptoms increased with 12.71 (delta 1) and 13.74 (delta 2) for patients undergoing APS with axillary radiotherapy compared to APS alone, while arm symptoms increased with 6.85 (delta 1) and 7.66 (delta 2) for patients undergoing ALND compared to APS alone. This suggests that patients who undergo APS with axillary radiotherapy may experience a greater increase in arm symptoms than patients undergoing an ALND. However, it is important to note that the comparison is not directly between APS with axillary radiotherapy and ALND. Therefore, more research is necessary to determine whether the difference in arm symptoms is statistically significant between these two axillary treatments.

### Strengths and limitations

One of the main strengths of this study is its relatively large sample size and the availability of a wide range of patient, treatment, and tumour characteristics that were collected from medical records and used in the regression analysis. Furthermore, there are no previous studies that have compared HRQoL as measured by the BREAST-Q or EORTC QLQ-BR23 between the four different axillary treatment groups (including axillary radiotherapy). This makes this research valuable for future comparisons. Another major strength is the longitudinal design of the study. Baseline and postoperative HRQoL outcomes were included, which allowed for the observation of the effect of different axillary treatments on HRQoL over time while correcting for clinically relevant variables. Additionally, by including baseline HRQoL measurements, it was possible to account for any potential missing patient history.

An important limitation is the fact that patients were not randomly assigned to a treatment group since there are specific treatment protocols for each breast cancer stage [11]. This may lead to confounding by indication. Moreover, most of the baseline characteristics differed significantly between treatment groups. By including various patient and treatment characteristics in the mixed models, it was tried to correct for as many confounders as possible. Especially the correction for neo-adjuvant chemotherapy was important, as patients undergoing a RISAS procedure always undergo neoadjuvant chemotherapy in contrast to patients undergoing an SLND and because chemotherapy affects HRQoL [48]. There may be residual confounding since it is not possible to control for unknown confounders. Furthermore, there was no data on for example socioeconomic status, educational level, and coping strategies, which can also have an impact on HRQoL [49, 50]. Additionally, the non-responder analysis revealed similar distribution patterns of baseline characteristics among the treatment groups, except for BMI. Non-responders tended to have a higher BMI compared to responders. Although BMI is linked to HRQoL, its influence on the overall outcomes is not expected to be significant since the non-responder subgroup accounts for less than 5% of the total sample size [51]. Lastly, there was a relatively high level of missingness per timepoint. This may be caused by patients switching hospitals or lacking motivation to complete PROMs. In addition, the sexual wellbeing domain of the BREAST-Q was not mandatory, resulting in even more missingness for this specific domain. The substantial missingness of data, especially in the BREAST-Q domains, may impact the validity of the results and may introduce bias. Therefore, it is recommended to validate the current results in other breast cancer cohorts.

### Future perspectives

In the future, it would be beneficial to conduct a validation study utilizing a multicentre database such as the Dutch MINIMAX study to compare HRQoL among patients receiving APS with axillary radiotherapy and patients receiving ALND alone [52]. Those results will be of great value to both patients and healthcare providers especially when there is a choice between treatments with comparable oncological outcome. Moreover, it would be interesting to examine HRQoL at intervals of 2- or 5-years postoperatively to determine if there are any changes in HRQoL between treatment groups. Radiation fibrosis should then be included in the analysis as it typically occurs 12 months after treatment and significantly impacts HRQoL [53–55]. Additionally, the use of more fixed HRQoL outcomes defined in core outcome sets is recommended to make more meaningful comparisons in future studies.

The findings of this study will be informative for healthcare providers and breast cancer patients in the future as they will enhance expectation management and shared decision making. In addition, this study offers patients perspective and demonstrates that the decline in their HRQoL may stabilize again after six months. This has the potential to motivate patients to complete PROMs, which may result in more HRQoL data for future research. Furthermore, this study provides a foundation for further research on the impact of axillary treatment on HRQoL, as axillary treatment is an emerging area of research [16, 56–60]. Once the oncological safety of less invasive and less extensive axillary treatments has been evaluated and protocols may be adjusted, this study can be used to develop predictive models and decision-making tools for patients.

## Conclusion

Physical and psychosocial wellbeing decreased significantly in patients receiving ALND with axillary radiotherapy compared to patients receiving APS alone. Patients undergoing APS without axillary radiotherapy had significantly fewer arm symptoms compared to patients receiving APS with axillary radiotherapy and patients receiving ALND with or without axillary radiotherapy. Shared decision making and expectation management could be strengthened by discussing arm symptoms per axillary treatment with the patient.

### Supplementary Information

Below is the link to the electronic supplementary material.

Supplementary file1 (DOCX 20 kb)

## Data Availability

The datasets generated during and/or analysed during the current study are not publicly available due to privacy or ethical restrictions but are available from the corresponding author on reasonable request.
