# Supplementary material for: Health-related quality of life (HRQoL) after different axillary treatments in women with breast cancer: a 1-year longitudinal cohort study
Source: Qual Life Res. 2023 Oct 27;33(2):467–79. doi: 10.1007/s11136-023-03538-3 (PMC10850260; doi:10.1007/s11136-023-03538-3)
Supplement: Supplementary file 1 — Supplementary file1 (DOCX 20 kb) [file 11136_2023_3538_MOESM1_ESM.docx]

**Appendix 1**

Table 4 Non-responder analysis

| **Variables** | **Responders**  **(n = 552)** | **Non-responders**  **(n = 27)** | **p-value** |
| --- | --- | --- | --- |
| *Axillary treatment* |  |  |  |
| APS | 386 (69.9%) | 15 (55.6%) | 0.070 |
| ALND | 59 (10.7%) | 3 (11.1%) |  |
| APS + Rtx | 39 (7.1%) | 1 (3.7%) |  |
| ALND + Rtx | 68 (12.3%) | 8 (29.6%) |  |
| *Age* |  |  |  |
| Mean (SD) | 53.7 (14.3) | 49.2 (14.3) | 0.119 |
| *BMI* |  |  |  |
| <25 | 255 (46.2%) | 11 (40.7%) | **0.008** |
| 25-30 | 213 (38.6%) | 6 (22.2%) |  |
| >30 | 84 (15.2%) | 10 (37.0%) |  |
| *Gene mutation* |  |  |  |
| No mutation | 219 (39.7%) | 10 (37.0%) | 0.841 |
| BRCA 1 mutation | 40 (7.2%) | 3 (11.1%) |  |
| BRCA 2 mutation | 27 (4.9%) | 2 (7.4%) |  |
| Other gene mutation | 11 (2.0%) | 1 (3.7%) |  |
| Unknown | 255 (46.2%) | 11 (40.7%) |  |
| *Bilateral cancer* |  |  |  |
| Yes | 27 (4.9%) | 0 (0%) | 0.478 |
| No | 525 (95.1%) | 27 (100%) |  |
| *Surgery* |  |  |  |
| Mastectomy | 161 (29.2%) | 6 (22.2%) | 0.475 |
| BCS | 278 (50.4%) | 13 (48.1%) |  |
| Mastectomy + reconstruction | 113 (20.5%) | 8 (29.6%) |  |
| *Receptor* |  |  |  |
| Triple negative | 85 (15.4%) | 8 (29.6%) | 0.053 |
| HER2 positive | 58 (10.5%) | 5 (18.5%) |  |
| HR positive & HER2 negative | 366 (66.3%) | 14 (51.9%) |  |
| Unknown | 43 (7.8%) | 0 (0%) |  |
| *T-stage* |  |  |  |
| pT0 | 53 (9.6%) | 3 (11.1%) | 0.294 |
| pT1 | 311 (56.3%) | 15 (55.6%) |  |
| pT2 | 110 (19.9%) | 9 (33.3%) |  |
| pT3 | 17 (3.1%) | 0 (0%) |  |
| pT4 | 2 (0.4%) | 0 (0%) |  |
| pTis | 59 (10.7%) | 0 (0%) |  |
| *N-stage* |  |  |  |
| pN0 | 422 (76.4%) | 18 (66.7%) | 0.167 |
| pN1 | 103 (18.7%) | 5 (18.5%) |  |
| pN2 | 20 (3.6%) | 3 (11.1%) |  |
| pN3 | 7 (1.3%) | 1 (3.7%) |  |
| *Neoadjuvant CTx* | |  |  |
| No | 397 (71.9%) | 16 (59.3%) | 0.229 |
| Yes | 155 (28.1%) | 11 (40.7%) |  |
| *Adjuvant CTx* | |  |  |
| No | 416 (75.4%) | 19 (70.4%) | 0.720 |
| Yes | 136 (24.6%) | 8 (29.6%) |  |
| *Neoadjuvant HTx* | |  |  |
| No | 531 (96.2%) | 26 (96.3%) | 1 |
| Yes | 21 (3.8%) | 1 (3.7%) |  |
| *Adjuvant HTx* |  |  |  |
| No | 264 (47.8%) | 14 (51.9%) | 0.832 |
| Yes | 288 (52.2%) | 13 (48.1%) |  |
| *Breast/chest Rtx* | |  |  |
| No | 195 (35.3%) | 9 (33.3%) | 0.811 |
| Yes | 331 (60.0%) | 16 (59.3%) |  |
| Yes & parasternal | 26 (4.7%) | 2 (7.4%) |  |
| *Rtx boost* |  |  |  |
| No | 415 (75.2%) | 17 (63.0%) | 0.231 |
| Yes | 137 (24.8%) | 10 (37.0%) |  |

**Appendix 2**

*Table 5 Extra HRQoL scores on all follow-up times according to treatment group*

| **HRQoL** | **APS**  **(n=386)** | **ALND**  **(n=59)** | **APS + Rtx**  **(n=39)** | **ALND + Rtx**  **(N=68)** | **p-value** |
| --- | --- | --- | --- | --- | --- |
| *BREAST-Q: Satisfaction with breasts at baseline* | |  |  |  |  |
| Mean (SD) | 71.6 (21.9) | 67.8 (19.7) | 73.6 (16.1) | 73.6 (22.1) | 0.521 |
| Missing | 89 (23.1%) | 15 (25.4%) | 7 (17.9%) | 14 (20.6%) |  |
| *BREAST-Q: Satisfaction with breasts at 6 months* | | |  |  |  |
| Mean (SD) | 69.5 (20.5) | 64.9 (19.7) | 71.0 (17.6) | 62.5 (20.0) | **0.040** |
| Missing | 96 (24.9%) | 12 (20.3%) | 10 (25.6%) | 15 (22.1%) |  |
| *BREAST-Q: Satisfaction with breasts at 12 months* | | |  |  |  |
| Mean (SD) | 69.8 (21.6) | 58.7 (23.7) | 65.6 (22.1) | 61.2 (18.3) | **<0.001** |
| Missing | 84 (21.8%) | 9 (15.3%) | 7 (17.9%) | 16 (23.5%) |  |
| *EORTC QLQ-BR23: Body image at baseline* | |  |  |  |  |
| Mean (SD) | 90.8 (15.6) | 85.2 (21.8) | 87.4 (17.9) | 79.9 (26.0) | **0.015** |
| Missing | 88 (22.8%) | 15 (25.4%) | 6 (15.4%) | 10 (14.7%) |  |
| *EORTC QLQ-BR23: Body image at 6 months* | |  |  |  |  |
| Mean (SD) | 81.3 (23.8) | 70.2 (27.7) | 79.4 (19.9) | 71.4 (27.1) | **0.002** |
| Missing | 89 (23.1%) | 12 (20.3%) | 9 (23.1%) | 12 (17.6%) |  |
| *EORTC QLQ-BR23: Body image at 12 months* | |  |  |  |  |
| Mean (SD) | 82.8 (23.5) | 71.2 (27.3) | 83.9 (18.6) | 74.1 (26.6) | **0.001** |
| Missing | 73 (18.9%) | 9 (15.3%) | 7 (17.9%) | 15 (22.1%) |  |
| *EORTC QLQ-BR23: Sexual functioning at baseline* | | |  |  |  |
| Mean (SD) | 24.5 (23.5) | 17.8 (21.6) | 30.6 (25.9) | 26.4 (26.5) | 0.142 |
| Missing | 94 (24.4%) | 16 (27.1%) | 9 (23.1%) | 10 (14.7%) |  |
| *EORTC QLQ-BR23: Sexual functioning at 6 months* | | |  |  |  |
| Mean (SD) | 26.2 (23.3) | 21.6 (23.5) | 20.7 (25.5) | 21.0 (21.0) | 0.188 |
| Missing | 101 (26.2%) | 12 (20.3%) | 10 (25.6%) | 14 (20.6%) |  |
| *EORTC QLQ-BR23: Sexual functioning at 12 months* | | |  |  |  |
| Mean (SD) | 27.4 (24.1) | 25.8 (23.8) | 21.8 (21.9) | 25.5 (23.2) | 0.697 |
| Missing | 87 (22.5%) | 10 (16.9%) | 10 (25.6%) | 17 (25.0%) |  |
| *EORTC QLQ-BR23: Sexual enjoyment at baseline* | | |  |  |  |
| Mean (SD) | 30.6 (36.2) | 23.7 (34.5) | 40.4 (42.3) | 29.3 (36.5) | 0.345 |
| Missing | 84 (21.8%) | 14 (23.7%) | 6 (15.4%) | 10 (14.7%) |  |
| *EORTC QLQ-BR23: Sexual enjoyment at 6 months* | |  |  |  |  |
| Mean (SD) | 30.1 (35.6) | 23.6 (29.9) | 21.5 (32.8) | 28.0 (33.5) | 0.435 |
| Missing | 87 (22.5%) | 11 (18.6%) | 8 (20.5%) | 12 (17.6%) |  |
| *EORTC QLQ-BR23: Sexual enjoyment at 12 months* | | |  |  |  |
| Mean (SD) | 31.0 (37.5) | 26.8 (33.3) | 33.3 (37.9) | 23.9 (32.3) | 0.652 |
| Missing | 73 (18.9%) | 8 (13.6%) | 7 (17.9%) | 15 (22.1%) |  |
| *EORTC QLQ-BR23: Future perspective at baseline* | | |  |  |  |
| Mean (SD) | 58.4 (28.2) | 48.1 (33.8) | 57.6 (32.6) | 47.1 (36.4) | 0.054 |
| Missing | 87 (22.5%) | 14 (23.7%) | 6 (15.4%) | 10 (14.7%) |  |
| *EORTC QLQ-BR23: Future perspective at 6 months* | | |  |  |  |
| Mean (SD) | 67.3 (26.7) | 53.9 (33.7) | 58.1 (28.5) | 51.8 (33.0) | **<0.001** |
| Missing | 88 (22.8%) | 12 (20.3%) | 8 (20.5%) | 12 (17.6%) |  |
| *EORTC QLQ-BR23: Future perspective at 12 months* | | |  |  |  |
| Mean (SD) | 68.1 (26.5) | 60.8 (31.8) | 63.5 (30.9) | 55.3 (32.0) | **0.024** |
| Missing | 74 (19.2%) | 8 (13.6%) | 7 (17.9%) | 15 (22.1%) |  |
| *EORTC QLQ-BR23: Systemic therapy at baseline* | | |  |  |  |
| Mean (SD) | 8.34 (11.0) | 12.2 (12.0) | 12.4 (18.7) | 17.8 (21.2) | **<0.001** |
| Missing | 85 (22.0%) | 14 (23.7%) | 6 (15.4%) | 11 (16.2%) |  |
| *EORTC QLQ-BR23: Systemic therapy at 6 months* | | |  |  |  |
| Mean (SD) | 16.0 (14.3) | 20.8 (16.4) | 19.4 (15.8) | 22.4 (17.4) | **0.018** |
| Missing | 89 (23.1%) | 12 (20.3%) | 8 (20.5%) | 12 (17.6%) |  |
| *EORTC QLQ-BR23: Systemic therapy at 12 months* | | |  |  |  |
| Mean (SD) | 13.1 (12.8) | 14.2 (12.4) | 16.1 (12.9) | 17.6 (15.1) | 0.115 |
| Missing | 73 (18.9%) | 8 (13.6%) | 7 (17.9%) | 15 (22.1%) |  |
| *EORTC QLQ-BR23: Hair loss at baseline* | |  |  |  |  |
| Mean (SD) | 1.88 (9.82) | 6.82 (18.4) | 1.01 (5.80) | 8.05 (21.9) | **0.002** |
| Missing | 85 (22.0%) | 15 (25.4%) | 6 (15.4%) | 10 (14.7%) |  |
| *EORTC QLQ-BR23: Hair loss at 6 months* | |  |  |  |  |
| Mean (SD) | 6.15 (16.3) | 9.93 (25.9) | 8.60 (22.7) | 7.14 (19.8) | 0.989 |
| Missing | 88 (22.8%) | 12 (20.3%) | 8 (20.5%) | 12 (17.6%) |  |
| *EORTC QLQ-BR23: Hair loss at 12 months* | |  |  |  |  |
| Mean (SD) | 4.15 (14.1) | 3.92 (15.8) | 5.21 (14.9) | 2.52 (8.89) | 0.865 |
| Missing | 73 (18.9%) | 8 (13.6%) | 7 (17.9%) | 15 (22.1%) |  |
| *EORTC QLQ-BR23: Breast symptoms at baseline* | | |  |  |  |
| Mean (SD) | 13.0 (14.9) | 14.4 (13.8) | 9.09 (9.63) | 13.2 (16.5) | 0.546 |
| Missing | 84 (21.8%) | 14 (23.7%) | 6 (15.4%) | 10 (14.7%) |  |
| *EORTC QLQ-BR23: Breast symptoms at 6 months* | | |  |  |  |
| Mean (SD) | 20.9 (17.9) | 17.2 (14.1) | 22.3 (20.5) | 22.8 (17.2) | 0.535 |
| Missing | 88 (22.8%) | 11 (18.6%) | 8 (20.5%) | 12 (17.6%) |  |
| *EORTC QLQ-BR23: Breast symptoms at 12 months* | | |  |  |  |
| Mean (SD) | 17.3 (18.9) | 17.2 (21.6) | 14.8 (17.4) | 18.2 (15.8) | 0.527 |
| Missing | 73 (18.9%) | 8 (13.6%) | 7 (17.9%) | 15 (22.1%) |  |
